# Current State and Future Perspectives on Personalized Metabolomics

**DOI:** 10.3390/metabo13010067

**Published:** 2023-01-01

**Authors:** Oxana P. Trifonova, Dmitry L. Maslov, Elena E. Balashova, Petr G. Lokhov

**Affiliations:** Institute of Biomedical Chemistry, 10 Building 8, Pogodinskaya Street, 119121 Moscow, Russia

**Keywords:** personalized metabolomics, mass spectrometry, blood plasma, diagnostics

## Abstract

Metabolomics is one of the most promising ‘omics’ sciences for the implementation in medicine by developing new diagnostic tests and optimizing drug therapy. Since in metabolomics, the end products of the biochemical processes in an organism are studied, which are under the influence of both genetic and environmental factors, the metabolomics analysis can detect any changes associated with both lifestyle and pathological processes. Almost every case-controlled metabolomics study shows a high diagnostic accuracy. Taking into account that metabolomics processes are already described for most nosologies, there are prerequisites that a high-speed and comprehensive metabolite analysis will replace, in near future, the narrow range of chemical analyses used today, by the medical community. However, despite the promising perspectives of personalized metabolomics, there are currently no FDA-approved metabolomics tests. The well-known problem of complexity of personalized metabolomics data analysis and their interpretation for the end-users, in addition to a traditional need for analytical methods to address the quality control, standardization, and data treatment are reported in the review. Possible ways to solve the problems and change the situation with the introduction of metabolomics tests into clinical practice, are also discussed.

## 1. Introduction

Personalized medicine is based on the importance of the individuals’ characteristics for early disease diagnostics and the positive response to treatment. According to the P4 concept that medicine should be preventive, predictive, personalized, and participatory, this may be achieved by the application of the tools and strategies of the systems biology in clinics. Using the global, integrative and dynamic approaches, and the big data sets analyses, the personalized medicine could provide deep insights into disease mechanisms that stratifies complex diseases into subtypes and discovers new approaches to drug targeting, and makes possible both the disease diagnostics and health assessment of an individual, by a universal noninvasive biological sample, such as blood [1,2]. Current clinical practice deals with a limited number of physiological parameters and thus is based on small amounts of information of the organism’s state. Modern post genomic technologies allow to perform a comprehensive analysis of the organism at various biological organization levels, from the genes to metabolites, providing new ways for the treatment and prevention of diseases, allowing for an early diagnosis and increasingly targeted pharmacological treatments [3,4]. Metabolomics is the youngest omics, following proteomics, and is often considered as the most promising for clinical practice. Metabolomics studies metabolites—both endogenous and exogenous low molecular weight compounds (up to 1000–1500 Da), which can be as substrates, as final products of the biochemical processes in the organism. Therefore, the metabolome, as the total of all metabolites, reflects as the internal pathophysiological processes in the organism, as an effect of the environment [5,6]. The Metabolomics Society has declared that “the narrow range of chemical analyses in current use by the medical community today will be replaced in the future by analyses that reveal a far more comprehensive metabolic signature. This signature is expected to describe global biochemical aberrations that reflect patterns of variance in states of wellness, more accurately describe specific diseases and their progression, and greatly aid in differential diagnosis” [7].

**Figure 1:**
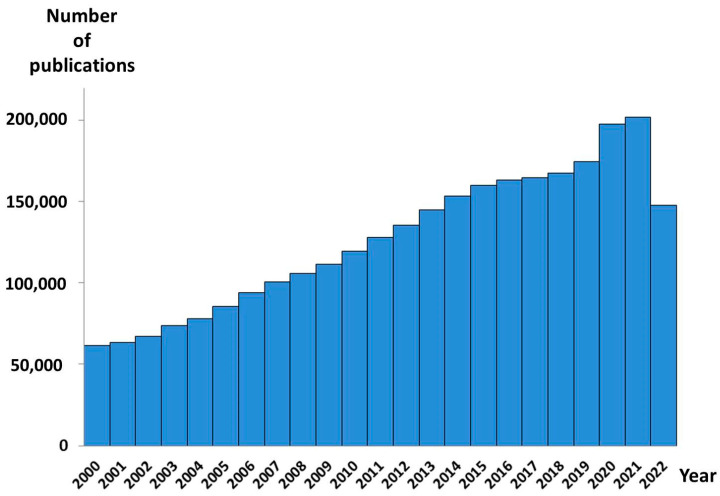
Number of metabolomics-based disease diagnostics-related publications indexed in PubMed from 2000 to 2022. The PubMed database was accessed (November 2022) with the keywords—metabolomic OR metabolome AND disease diagnostic OR disease diagnosis.

**Figure 2:**
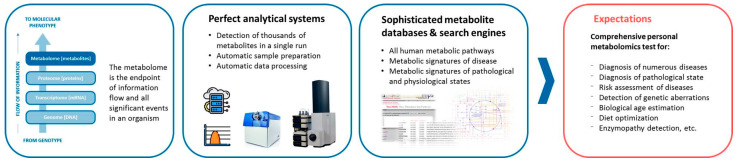
Advantages of metabolomics that determine its promising perspectives for clinical diagnostics.

According to PubMed, more than 3,000,000 metabolomic studies aimed at discovering new disease diagnostics have been published to date. More than 2,500,000 were published since 2000. By early 2007, the annual number of such papers has exceeded 100,000 (Figure 1). Each new study provides new data on the diseases’ mechanisms, drug targets, and therapy, moving us one step closer to discovering the omics-tests proper for clinical implementation [8,9,10]. The metabolomic data accumulated over the last decades in the appropriated databases contain comprehensive information about more than 200,000 metabolite entries, more than 800 human metabolic pathways, metabolite sets, and abnormal concentrations of metabolites associated with different conditions and diseases, and descriptions of metabolite locations in the organs, tissues, and even their subcellular localization. Hundreds of disease metabolite signatures detected in the human blood, urine, cerebral spinal fluid, and feces, are presented [11]. Thus, the use of metabolomic data, along with the high-throughput measurements of the large sets of low-molecular-weight substances in biosamples, make it possible to implement personalized metabolomics into clinical practice (Figure 2) [12]. However, despite such huge data sets collected to date, and such promising perspectives of personalized metabolomics, there are no Food and Drug Administration (FDA)-approved metabolomics tests yet. In this review, we discuss the well-known problem of the complexity of the personalized metabolomics data analyses and their interpretation for the end-users, in addition to a traditional need for analytical methods to address quality control, standardization, and data treatment, and possible ways to solve the problems. 

## 2. The Bottle Necks of Personalized Metabolomics

### 2.1. Preanalitical and Analytical Methods

**Figure 3:**
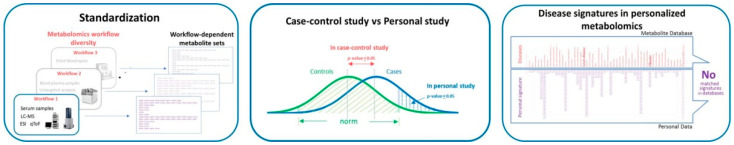
Challenges of personalized metabolomics. Standardization represents the workflow-dependent challenge, when the sets of detected metabolites (including the metabolic signatures of diseases) correspond to the protocols used for the metabolomic studies, which are diverse. Case-control studies compare the group characteristics and a metabolite detected in a case-control metabolomic study, as case-associated (*p* < 0.05), may not be considered out of the norm in a personalized metabolomics study. The problem of no matched signatures, in a case of personal data searching against a metabolic database, is due to the medley of fragmented signatures in the personalized metabolomics data (for details please see [12]).

There are several recent reviews that discuss why the results of numerous successful metabolomic studies have not yet seen their new diagnostics tests implemented into clinical practice [9,10,12,13,14,15,16,17,18,19]. All of them have reported that the pipeline of the biomarker development includes several key stages, consisting of discovery, validation, and the clinical translation of the finds. At each of these stages, the progress has been achieved, in terms of the technological advances for the production, analysis, and sharing of the metabolomics data, but some limitations still exist. Figure 3 illustrates the challenges in answering the question of why, despite all promising perspectives for the implementation of metabolomics into clinical diagnostics, it has not been yet happened. The challenges are all related to the differences between metabolomics, as an exploratory study, rather than metabolomics, as a personalized diagnosis. The main difference is that the metabolomics study is usually a case-control type (group vs. group) and a personal analysis compares a sample with a control set. The personalized metabolomics analysis should be scalable, fast, and understandable to wider range of people. While a metabolomics study is unique, it can take from several months to several years, and with very complex results that are intended for scientists with experience in the same field of science [12,20]. Figure 3 below shows the traditional needs for the analytical methods to address both the metabolomics study and personalized metabolomics, with a focus on the differences, including the standardization and treatment of data.

In 2007, the Metabolomics Society launched the Metabolomics Standards Initiative (MSI) Committee for the development of quality control and standard operating procedures, which should be carefully followed throughout the research process [7,21,22,23,24]. It is well known that the main problem of all methods developed for diagnostic purposes is that standardization is needed during all of the process stages, initiating from the criteria of the studied cohorts selection, the method of the metabolomics analysis used, and ending with the statistical analysis of the data. the standardization of all of these steps allows for the prevention of the risk of poor-quality control metabolomics protocols, incorrect quantification of metabolites, and deceptive data interpretation [25,26,27,28,29,30,31].

For example, the various metabolomic studies of the same disease can give dramatically different or only partly similar results, because of the differences in the study design, including the principles of the studied group formation, as well as of the participants’ differences, such as age, sex, disease duration, age of disease onset, and presence of other comorbidities or risk factors for the disease progression. The mentioned discrepancies between the studies indicate a need of a high quality, well-thought-out experimental design, including the careful consideration of the studied cohorts and the selection of the appropriate control individuals. Of course in a case of blood, the sample must be collected in a fasting state, to minimize unwanted sources of variability on the metabolome. To reduce the effect of inter- and intra-individual variations, the analyzed groups should be similar in demographic (gender, ethnicity, age), lifestyle (diet), and physiological factors (body mass index (BMI)), in addition to the parameters directly related to the aim of the study. The appropriate data should be carefully collected and can be used, not only for the experiment design, but for the data analysis [7]. In the case of an expected high inter-individual variability, a larger sample cohort could even be required. The sample size is one of the main factors affecting the experimental results and should be adequate to provide the statistically robust investigation, taking into account the phenotypic variation in the metabolome. The number of samples needed to fully identify and understand the mechanisms of a disease, may depend on the complexity of a disorder [32]. Very often, the limitation of the metabolomics study is small sized groups and the absence of a validation cohort that can lead to a lack of statistical robustness and validity of the results [33]. 

The following problem is in the detected metabolite sets that are workflow-dependent because they were obtained using different analytical platforms, and so the experimental setups were different in the sample preparation routines and measuring equipments. That is why, very often, the detected sets of metabolites are specific for the particular study. 

According to the existing databases, the human metabolome contains thousands of metabolites differing in their concentrations (from g/L to pg/L), chemical and physical properties, and stability [24]. Therefore, different analytical platforms and different sample preparation procedures are needed to analyze the metabolome comprehensively [34,35]. Currently, there are no standardized metabolite extraction protocols and it is usually selected depending on the metabolites of interest [36]. The same situation is observed for the instrumental techniques. The use of 1H nuclear magnetic resonance (NMR) spectroscopy and mass spectrometry (MS) are the two main analytical techniques in metabolomics. Both of them can be used for the identification and quantification of a large number of metabolites in complex biosamples. In spite of NMR, it is characterized by a better reproducibility, MS-based techniques have a higher sensitivity. Therefore MS-based technology is widely used in the clinically-oriented metabolomic research. Regardless of whether we use untargeted or targeted metabolomics methods, both approaches have the same pitfalls, due to the diversity of the metabolome [37,38]. In both cases, only a particular class of compounds as chemical or physical properties can be measured, due to the sample preparation protocol, separation method (gas or liquid chromatographic separation, one or two dimensional), and the mass spectrometry ionization method used in the study. For example, the gas chromatography-mass spectrometry (GC-MS) method can analyze only volatile metabolites or those that can be volatilized, such as most amino acids, sugar alcohols, aromatic amines, and organic acids [39,40]. The liquid chromatography-mass spectrometry (LC-MS) method is able to analyze both polar and non-polar compounds of different classes, by using different chromatographic columns [41,42,43]. The direct injection mass spectrometry (DIMS) method can analyze only abundant metabolites, limited by the concentration range of the detector, but it is faster and has a better reproducibility than the “hyphenated” techniques [44,45]. 

Thus, each metabolomics study does not research the metabolome itself, but the possibilities of the analytical approach used. Therefore, in our view, to date, there is no technique allowing for the analysis of the whole metabolome to capture the personalized metabolomic profile. At the same time, a possible alternative option using a combination of different techniques, would lead to increased time and cost of the analysis, as well as the complicated data interpretation for the end-users. Furthermore, the personalized metabolomic test cannot be delivered as an aggregation of the variety of experiments performed in different conditions.

### 2.2. Data Processing and Interpretation 

The data obtained in the metabolomic studies are complex, in terms of the number of parameters measured, and a robust statistical analysis of the results is needed. Classical (*t*-tests, ANOVA, and a nonparametric Mann–Whitney–Wilcoxon test) and a multi-variate (principal component analysis (PCA), hierarchical cluster analysis (HCA), and partial least square-discriminant analysis (PLSDA)) statistical methods are usually used for a reliable analysis in metabolomic studies [46]. Because of the multiple-testing issue, the Bonferroni correction and the false discovery rate (FDR) approaches are needed to limit the false positive data, especially in untargeted metabolomics [47]. There are free web-based platforms for mass spectrometry-based metabolomics data processing and the following analysis, such as MetaboAnalyst [48], XCMS Online [49], or PAIRUP-MS [50], which enable analyses of the raw data for the biomarker search and pathway enrichment analysis. Unfortunately, there are no web-based platforms for a personalized metabolomics analysis, and most of the widely used statistical workflows are only possible for the case-control studies, and they do not work at the individual level due to the intra- and inter-individual biological variabilities. In addition, in the case of a personalized analysis, the samples usually cannot be analyzed in a single batch and the technical variations should be taken into account during the data processing. 

The metabolite identification is one of the key steps of any metabolomics study and is also critical for pathway analysis and mapping [51]. The metabolome databases—Human Metabolome Database (HMDB) (http://www.hmdb.ca, accessed on 30 November 2022) [11], FooDB (http://foodb.ca, accessed on 30 November 2022), DrugBank (https://go.drugbank.com, accessed on 30 November 2022), and Toxin and Toxin Target Database (T3DB) (http://www.t3db.ca, accessed on 30 November 2022) [52], with spectral libraries—Metlin (https://metlin.scripps.edu, accessed on 30 November 2022) [53] or mzCloud (https://www.mzcloud.org, accessed on 30 November 2022), contain data on thousands of metabolites, including experimental and modeling data. However, not all metabolites are annotated already. In contrast to proteomics, the metabolomics data are repositored in numerous databases and usually in an ununified data format. Most of metabolomics data are accumulated in the published papers and cannot be used for data analysis directly [14,54,55]. The metabolome cannot be predicted, as the proteome. In a case of proteins, the identification analysis of the mass spectrometry data is performed, based on the previously determined data about the protein encoding genes in the existing databases. The protein sequence can be predicted using the genome data, and so their identification is based on the previous more stable omics level. The metabolome cannot be predicted at all as it reflects the effect of both the internal and external factors on an organism. A metabolomics analysis enables the detection of any variation associated with both differences in lifestyle (diet, physical activity, and use of drugs or supplements) of various individuals (inter-individual variations) and changes occurring in the lifestyle of an individual (intra-individual variations). That is why the metabolome, as the set of final products of biochemical processes in an organism, is more flexible and better reflects the actual state of the individual, than the genome and proteome, but it cannot be identified so simply.

The accurate identification of the metabolites in complex biosamples requires MS platforms with a high mass resolution (>50,000, M/ΔM, full width at half maximum) and a high mass measurement accuracy (<3 ppm) for the isotope pattern detection, especially in the case of metabolites with similar structures and overlapping MS peaks. The accurate retention time and the MS/MS spectra increase the identification confidence by matching with the reference data in the spectral libraries. However, instruments, fragmentation conditions, and types of molecules, can be different in various databases. Therefore, the most confident mass spectra for the definitive identification of a metabolite are those obtained from a pure chemical standard at the same MS platform. Unfortunately it is limited by the availability of such standards, especially commercial [56,57,58,59]. In proteomics, it is not difficult to synthesize the standard, if the peptide sequence is known, while in metabolomics, it is challenging, due to large structure diversity. For example, the differences in the hydrocarbon chains of fatty acids that are present in phospholipids, can differ in the exact individual fatty acyl groups present, the positional distribution of the fatty acyl groups, the double bond location, geometry, etc. In contrast to the proteins, where fragmentation patterns may be predicted, based on the amino acids sequence, metabolites are fragmented in a relatively unpredictable manner and very often under low-energy conditions, or even in an ionization source. Although in the case of the personalized metabolomics analysis, there is no need to find new metabolites (biomarkers), the definitive identification of the already known compounds must be confirmed or the isotopically labeled standards should be used as the gold standard for the definitive metabolite identification [31].

The interpretation of the detected metabolite changes is challenging as well. Most of the biomarker studies aimed at the development of new diagnostic tests for clinics, deal with blood as minimally invasive and therefore use a more convenient biosample, especially for monitoring in large population groups. Blood interacts with all tissues and organs in the organism as well as with various cells, such as lymphocytes, macrophages, and leukocytes that contain proteins, metabolites, cell-free DNA and RNA arising by secretion, apoptosis, or enzymatic cleavage from cell membranes of intersecting organs. Thus, blood reflects the actual state of the organism and can serve as a “window” into health and disease [60]. However, despite the huge number of metabolomic studies that have been successful in discovering a disease associated the blood metabolome changes, the origin of the detected metabolite changes and thus, the mechanisms of a disease onset, stay unclear. Due to the non-organ specificity and the ability to reflect the biochemical processes of the whole organism, the interpretation of the changes detected in the blood is complicated [61,62]. In addition, all pathological processes in the organism are not the result of a single change in the metabolic pathway, but rather a coordinated change in more than one pathway. These changes may arise from a single change to a node involved in multiple pathways, or from multiple changes to the nodes in multiple pathways, as suggested by the complex mechanism of most diseases [63]. Therefore the findings about the role of the distinguished metabolites and the possible mechanisms of the disease onset or the progression can require additional research and the involvement of appropriate specialists.

It shouldn’t forgotten, that most of the metabolomic studies are case-control studies and their results cannot be directly translated to personalized metabolomics. In such studies, the individual features are not taken into account and are even filtered out from the data sets selected for the comparative analysis of the controls and cases. The potential biomarkers detected in this case are metabolites with a significantly different level in the samples of the studied groups. The experience shows that a single biomarker cannot always allow for the distinction between the case from the control and the combination of biomarkers (metabolite signature), summarizing their diagnostic potential that provides a better performance [64,65,66]. However, the detection of the signature in the personal data is challenging. Even more, the metabolite with statistically significant differences between groups can have a concentration outside of the norm in only a small part of the samples in the case group. In such a case, the metabolic signature consisting of such metabolites should almost always give false-negative results for personal measurements [12,67].

Summarizing all of the mentioned above, it turns out that besides the challenges common for all metabolomic studies, the personalized metabolomics requires a completely new routine workflow, which has to be defined by taking into account the frequency of the analysis and the delivery time of the results to the end-users. The generally accepted methods of data processing and the knowledge collected in the metabolomic databases cannot be directly applied to the personalized metabolomics analysis. The known potential biomarkers and their signatures often deal with small concentration variations between the groups and so most likely correspond to the norm in the personalized analysis. This is the main reason why the accumulated data on thousands of metabolomic studies did not result in a revolution in the routine clinical care, in spite of the continuous improvement of the used analytical platforms.

### 2.3. Data Interpretation for the End-Users

**Figure 4:**
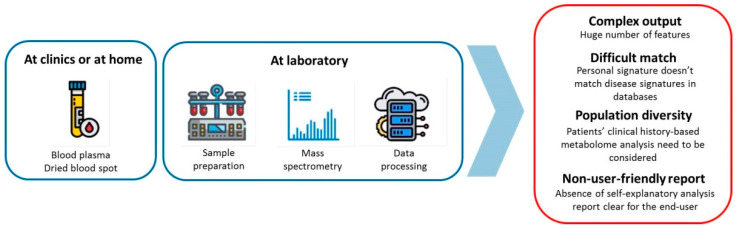
The main problems of personalized metabolomics workflow. Blood plasma or dried blood spot samples are collected at clinics or at home and transported to the laboratory. At the laboratory, after the sample preparation, the mass spectrometry analysis and the preliminary data processing of the final data analysis is challenging, due to the complex output, the difficult matching of the personal signature with the databases, the population diversity, and the absence of the algorithm to translate the complex metabolomics data into a user-friendly analysis report.

Today, there is still no workflow to perform the personalized metabolomics analysis that can be implemented in clinical practice. Current clinical practice deals with a limited number of physiological parameters based on a small amount of information of the organism state. The metabolomics provides the complex profile of the biosamples consisting of thousands of features, which reflects the actual state of the organism. Delivering the huge amount of measuring data in an understandable form, is challenging. Thus, the translation of the complex metabolomics data into a self-explanatory analysis report that is clear for the end-user, is a main problem for the personal metabolomics implementation (Figure 4). 

As the metabolome is influenced by both internal and external factors and reflects both the biochemical processes in an organism, including the pathological ones, and the effect of diet, drugs, and environment, it may be concerned as a mirror in which each individual can see their health state. Using modern techniques for analysis, such as for the sample collection, for example, the dried blood spot, provides the individual an opportunity to take care of their health by themselves. In this case, any slightest disturbance in the individual metabolome can be tracked in the real rhythm of life. It can be important for the assessment of the effect of life style, diet, physical activity, and drug supplementation on the organism. Ideally, in the future, the personalized metabolomics can replace instrumental methods of early disease diagnostics and save money and time that may be spent on the disease treatment. It well known that prevention is better than the cure. 

At the same time, the simple format of the metabolomics analysis data is crucial for clinicians, because they should make decisions and set diagnoses, based on confident analysis data. Today, the clinical diagnostics is based on several dozens of metabolites approved by the FDA and characterized by the concentration data in normal and diseased states. In other words, the existing clinical tests deliver the results in the report format allowing to diagnose a disturbance in the organism or propose additional tests at a glance. The main advantage of metabolomics is the ability to measure numerous metabolites in a biosample, simultaneously. However, the greater the number of parameters delivered in the analysis report, the greater the knowledge about the biology of the processes occurring in the organism, and the greater the amount of time for considering what will be required from the clinician to make a decision. [68]. Therefore the algorithm of the metabolomics data processing and interpretation to simplify the analysis report is a main bottleneck for the personalized metabolomics implementation in clinical practice. The development of such an algorithm would revolutionize laboratory diagnostics, and hundreds of discovered metabolic signatures would find a use in clinics. To this end, the metabolomics-based health data collection in families and large cohorts studies (e.g., well characterized biosamples from big biobanks) with electronic medical records for the integration of the biological information with the clinical data, can provide a basis for the personalized analysis. 

In conclusion to the section, it should be noted that it is difficult to determine the applicability of the common metabolomics tools for personalized metabolomics. Based on our experience in this field, the standard metabolomics tools cannot be directly applied for personal metabolomics. There is a feeling that additional data processing and in-house software are required for personal metabolomics.

## 3. Possible Ways of a Personalized Metabolomics Implementation

### 3.1. Multi-Omics Tests

The attempts to create and implement an omics-test for personalized medicine have existed for a long time, and various approaches have been used. One of the most popular is the multi-omics approach, including the genomics, transcriptomics, proteomics, and metabolomics analysis at the single-subject (N-of-1) studies, where they are used to analyze the biomaterial of a single person. Several recent studies have illustrated the utility of the multi-omics longitudinal data to look for signs of reversible early disease or disease risk factors in single individuals [69,70,71,72]. 

In 2012, Michael Snyder and colleagues presented the iPOP (integrated personal omics profiling) study [73]. It is a longitudinal study that combines genomic, transcriptomic, proteomic, metabolomic, and autoantibody profiles from a single individual of 109 participants over a 14 month period. A significant number of the iPOP participants are pre-diabetic, and a better understanding of the way that omics are influenced by a disease state and the progression to either a healthy or a diabetic state is another important focus of this study. Compiling invaluable omics-data with data on the participants’ diets, stress levels, activity levels, and personal and family medical history, allowed to better characterize a normal state of health on the molecular level, as well as to identify early signs of disease that may someday lead to the ability to better predict and treat diseases in the early stages, and perhaps even prevent disease altogether [74,75,76].

In 2014, Leroy Hood and Nathan D. Price proposed the 100,000 (100K) person wellness project, which was supported by the Arivale program in 2015 [77]. In the frame of this wellness program, the information for each participant over time, including the genomic, blood analytes, gut microbiome, and digital self-measurements, was collected and used by Arivale health coaches for providing participants a personalized list of recommendations for improving their wellness and avoiding disease. In 2017, the authors of the project presented the results of the Pioneer 100 Wellness Project (P100), based on the data of the whole genome sequences, clinical tests, metabolomes, proteomes, and microbiomes at 3-month intervals, and frequent activity measurements for 108 individuals over the course of 9 months [78]. In 2019, the Arivale wellness program was closed, but the collected data are still used for scientific purposes [79,80,81,82].

Due to integration of several omics technologies, the multi-omics approach is able to obtain additional knowledge about the individual but at the same time leads to theincrease of the cost and is time consuming, due to the complexity of the analysis. Furthermore, the main bottleneck of any multi-omics study is the analysis of the obtained data sets. A multi-omics approach requires an integration of multiple data types obtained at various molecular levels from the genome to metabolome, and their relation with the clinical data [64,83,84]. The population diversity and the lack of stated regulated procedures and standards for such omics-tests may be another reason for the slow translation of their findings to the medicine. The multi-omics initiatives point out that standard operating procedures (SOPs) for the standardization among methods and technical controls, in order to increase the results reproducibility and improve the reliability of the techniques, are needed [85]. That is why the results of the mentioned above N-of-1 studies have not yet received the widespread implementation as omics-tests. 

### 3.2. Laboratory Developed Tests

Another possible way to solve problems and implement the personalized metabolomics into clinical practice may be the laboratory-developed test (LDT) format. The LTD, as a subset of in vitro diagnostic devices (IVDs), has been widespread in clinical practice for decades [86,87,88,89]. IVDs are “those reagents, instruments, and systems intended for use in the diagnosis of a disease or other conditions, including a determination of the state of health, in order to cure, mitigate, treat, or prevent disease or its complication. Such products are intended for use in the collection, preparation, and examination of specimens taken from the human body” [90]. The LDT is defined by the US Food and Drug Administration (FDA) as “in vitro diagnostic tests that are manufactured by and used within a single laboratory”, i.e., a laboratory with a single clinical laboratory improvement amendments (CLIA) certificate. LDTs are also referred to as in-house developed tests or “homebrew” tests [91]. The concept of the LDT appeared in 1976, when the FDA had the authority to regulate IVDs as medical devices [92]. However, despite of the stringent FDA standards with robust regulatory processes of premarket validation for commercial IVDs, the they do not exist for LDTs, due to the limited availability and the primary use in the context of rare diseases. 

The LDT can measure both the individual and large number of analytes of various natures. Several omics-based LDTs have been published for the diagnosis of various diseases, including genetic disorders, cancer, and infections [93,94,95,96,97,98,99]. Only two papers have been found in PubMed through a search of “metabolomics AND LDT”. Both are from our laboratory and report about the metabolomics tests designed to diagnose early-stage Parkinson’s disease [100,101]. Currently there are no metabolomics-based LDTs in use. A number of LDTs were developed by Metabolon in 2018, but now there is no any information on the company website regarding whether these tests are still in use. Using the advanced metabolomics technologies, Metabolon has designed the Meta UDx™ test for the detection of abnormalities in major human metabolic pathways or biomarkers that cannot be measured by other means, and Meta IMD™ and Meta IMD™+ (Plus) tests for the diagnosis of rare genetic disorders, known as hereditary metabolic disorders or congenital metabolic disorders. These LDTs determined up to 1,000 metabolites in blood plasma and generated a heat map of the metabolite Z-scores, which were used to identify altered metabolic pathways, and were used in the CLIA certified laboratories. In addition, the company has offered a clinically confirmed quantose impaired glucose tolerance test, which used a proprietary algorithm to produce an “IGT score”, based on a combination of glucose and seven metabolites [102]. As LDTs, all of these tests have not been approved by the FDA and were used in the CLIA certified laboratories. Their results could be used in clinics as auxiliary tests and in combination with other standard clinical diagnostic tests [89]. 

Thus, using the LDT strategy, the advantages of metabolomics diagnostics have the potential to become a reality. In the LDT’s format, the implementation of the metabolomics-based tests is regulated by the protocols and standardization acts of particular laboratories and local rules for diagnostic devices only. Since the MS-based metabolomics analysis can be performed using dried blood spot samples, which can be collected by the individuals themselves and delivered to the laboratory by mail or courier service without special requirements for the transportation conditions, such as temperature, the LDT becomes convenient for customers and available almost everywhere [103]. In addition, this format is very useful for self-care, due to the ability to regularly monitor the state of the organism, which has a pronounced applied value in the modern world. Potentially, a large number of tests can be performed within one laboratory, and so the cost of the metabolomics LDT is expected to be quite low and acceptable for most people. 

It should be noted that alternative tools for monitoring metabolites in biological fluids, such as enzyme assays or immunoassays, have not been considered as a possible way of personal metabolomics implementation, due to their inconsistencies with the metabolome analysis concept itself. Such assays are useful for the analysis of a single metabolite, or possibly a set of particular metabolites, but suffer from low multiplexing capabilities. Thus, the tests, based on these methods cannot be used for a complex metabolomics analysis.

In conclusion to the section, it should be reported that in spite of all challenges, there are several examples that are beginning to use metabolomics as an individual health assessment. For example, Nightingale Health offers to estimate the “age you are likely to live to before falling ill from any of the top 10 diseases that significantly reduce the quality of life” from a single finger-prick blood sample taken at home [104]. Using the proprietary NMR-based blood analysis technology and software, the service gives an estimate of healthy years, based on the publications and the previous analysis results of hundreds of thousands of blood samples. Another one is AminoIndex^®^ Cancer Screening (AICS^®^) from the Ajinomoto Group Leveraging utilizing the LC/MS-based measurement of amino acids in plasma, to deliver a minimally invasive, early cancer detection [105]. The AminoIndex^®^ Cancer Screening (AICS^®^) system was introduced for stomach, lung, colorectal, pancreas, prostate, breast, uterine, and ovarian cancers at medical institutions in Japan. Although the AminoIndex^®^ service cannot be assigned as a real metabolomics test, due to the analysis of a limited number of metabolites, together with Nightingale Health, they can be considered as the successful example of the implementation of metabolomics to clinical practice and wellness. Furthermore, the Nightingale Health service confirms, one more time, that algorithms for data interpretation to self-explanatory analysis reports for the end-users is one of the key factors.

## 4. Conclusions

In spite of the challenges common for all metabolomic-based studies, the personalized metabolomics has a high chance to be implemented in clinical practice when the main problem will be resolved—the algorithm for the data interpretation to the self-explanatory analysis report for the end-users. Among the above mentioned strategies proposed by the professional community for developing an omics-test suitable for clinics, the LDT can be an actual possible way of personalized metabolomics implementation. Based on the current state of metabolomics and omics-based tests, the personalized metabolomics LDT has a great chance to be developed for the comprehensive diagnostics of human health. At first, the personalized metabolomics LDT meets the four key criteria for a viable market model: affordable price, availability of the test results to the end-user, a fast testing speed, and scalability. In addition, personalized metabolomics is potentially more convenient for the end-users, multi-functional, and more informative than existing clinical blood diagnostics.
